# Hijacking SARS-CoV-2/ACE2 Receptor Interaction by Natural and Semi-synthetic Steroidal Agents Acting on Functional Pockets on the Receptor Binding Domain

**DOI:** 10.3389/fchem.2020.572885

**Published:** 2020-10-23

**Authors:** Adriana Carino, Federica Moraca, Bianca Fiorillo, Silvia Marchianò, Valentina Sepe, Michele Biagioli, Claudia Finamore, Silvia Bozza, Daniela Francisci, Eleonora Distrutti, Bruno Catalanotti, Angela Zampella, Stefano Fiorucci

**Affiliations:** ^1^Department of Surgical and Biomedical Sciences, University of Perugia, Perugia, Italy; ^2^Department of Pharmacy, University of Naples Federico II, Naples, Italy; ^3^Net4Science S.r.l., University “Magna Græcia” of Catanzaro, Campus Universitario “S. Venuta”, Catanzaro, Italy; ^4^Microbiology Section, Department of Medicine, University of Perugia, Perugia, Italy; ^5^SC di Gastroenterologia ed Epatologia, Azienda Ospedaliera di Perugia, Perugia, Italy

**Keywords:** SARS-CoV-2, COVID-19, virtual screening, nutraceuticals, drug repurposing and repositioning, bile acids, spike protein

## Abstract

The coronavirus disease 2019 (COVID-19) is a respiratory tract infection caused by the severe acute respiratory syndrome coronavirus (SARS)-CoV-2. In light of the urgent need to identify novel approaches to be used in the emergency phase, we have embarked on an exploratory campaign aimed at repurposing natural substances and clinically available drugs as potential anti-SARS-CoV2-2 agents by targeting viral proteins. Here we report on a strategy based on the virtual screening of druggable pockets located in the central β-sheet core of the SARS-CoV-2 Spike's protein receptor binding domain (RBD). By combining an *in silico* approach and molecular *in vitro* testing we have been able to identify several triterpenoid/steroidal agents that inhibit interaction of the Spike RBD with the carboxypeptidase domain of the Angiotensin Converting Enzyme (ACE2). In detail, we provide evidence that potential binding sites exist in the RBD of the SARS CoV-2 Spike protein and that occupancy of these pockets reduces the ability of the RBD to bind to the ACE2 consensus *in vitro*. Naturally occurring and clinically available triterpenoids such as glycyrrhetinic and oleanolic acids, as well as primary and secondary bile acids and their amidated derivatives such as glyco-ursodeoxycholic acid and semi-synthetic derivatives such as obeticholic acid reduces the RBD/ACE2 binding. In aggregate, these results might help to define novel approaches to COVID-19 based on SARS-CoV-2 entry inhibitors.

## Introduction

The coronavirus disease 2019 (COVID-19) is a respiratory tract infection caused severe acute respiratory syndrome (SARS)-CoV-2, a newly emerged coronavirus first identified in the city of Wuhan in China in December 2019 (Zhu et al., 2020). Globally, as of June 9, 2020 there have been more than ~7 million confirmed cases of COVID-19, including 404,396 deaths (World Health Organization, 2020) in 216 countries (Fauci et al., 2020). Genetic sequencing SARS-CoV-2 demonstrates that the virus is a betacoronavirus sharing ~ 80% genetic identity with SARS-CoV and MERS-CoV, identified in 2003 and 2012, respectively, and ~ 96% identity with bat SARS-related CoV (SARS-CoV) RaTG13 (Wang et al., 2020b; Wrapp et al., 2020; Yan et al., 2020). Similarly to the 2003 and 2012 pandemics caused by these viruses (De Wit et al., 2016), the human infection caused by SARS-CoV-2 induces respiratory symptoms whose severity ranges from asymptomatic/poorly symptomatic to life threatening pneumonia and a cytokine related syndrome that might be fatal (Guan et al., 2020; Zou et al., 2020).

It is well-established that, similarly to SARS-CoV, SARS-CoV-2 enters the host cells by hijacking the human angiotensin converting enzyme receptor (ACE2) (Gui et al., 2017; Yuan et al., 2017; Walls et al., 2019, 2020; Wang et al., 2020b; Yan et al., 2020). The interaction of the virus with ACE2 is mediated by the transmembrane spike (S) glycoprotein, which shares 80% of the amino acid sequence identity with SARS-CoV and 97.2% of sequence homology with the bat SARS-CoV-RaTG13. In the case of SARS-CoV and SARS-CoV-2, the spike glycoprotein (S protein) on the virion surface mediates receptor recognition and membrane fusion (Lu et al., 2020). In the intact virus, the S protein assembles in a trimeric structure protruding from the viral surface. Each monomer of the trimeric S protein has a molecular weight of ≈180 kDa and contains two functional subunits, S1 and S2 that mediate, respectively, the attachment to ACE2 and the membrane fusion. The S1 binds to the carboxypeptidase domain of ACE2 with a dissociation constant (Kd) of ~15 nM (Hoffmann et al., 2020).

Structural analysis has demonstrated that the N- and C- terminal portions of S1 fold as two independent domains, N-terminal domain (NTD) and C-terminal domain (CTD), with the latter corresponding to the receptor-binding domain (RBD) (Wang et al., 2020b). According to the high-resolution crystal structure information available so far, the RBD moves like a hinge between two conformations (“up” or “down”) to expose or hide the residues binding the ACE2. Within the RBD, there is a receptor binding motif (RBM), containing two binding loops separated by a short β-sheet, which makes the primary contact with the carboxypeptidase domain of ACE2. Importantly, while amino acid alignment studies have shown that the RBD of SARS-CoV-2 shares 73.5% homology with SARS-CoV, the identity of RBM, the most variable region of RBD, is significantly lower (~50%) making it unclear whether the RBMs of the two viruses can induce cross-reactive antibodies. The region outside the RBM is thought to play an important role in maintaining the structural stability of the RBD.

The entry of SARS-CoV-2 in the host cells requires the cleavage of the S protein, a process that takes place in two steps. After binding to ACE2, the S protein is cleaved between the S1 and S2 subunits by a camostat-sensitive transmembrane serine protease, TMPRSS2 (Li et al., 2003; Lan et al., 2020; Shang et al., 2020; Wang et al., 2020b). Unlike SARS-CoV, SARS-CoV-2 has a distinct furin cleavage site (Arg-Arg-Ala-Arg) between the S1 and S2 domains, at residues 682–685, which may explain some of the biological differences. This furin cleavage site expands the versatility of SARS-CoV-2 for cleavage by cellular proteases and potentially increases the tropism and transmissibility owing to the wide cellular expression of furin proteases especially in the respiratory tract (Belouzard et al., 2009; Ou et al., 2020). Cleavage at the S1/S2 site is essential to unlock the S2 subunit, which, in turn, drives the membrane fusion. Importantly, a second S2 site of cleavage has been identified at the S2′ site which is thought essential to activate the protein for membrane fusion.

The spreading of the COVID-19 pandemic and the lack of effective therapies targeting the viral replication have prompted an impressive amount of investigations aimed at targeting several aspects of SARS-CoV-2 biology and viral interaction with ACE2. In this scenario, drug repurposing is a well-established strategy to quickly move already approved or shelved drugs to novel therapeutic targets, bypassing the time-consuming stages of drug development (Ghosh et al., 2020; Khan et al., 2020; Micholas and Jeremy, 2020). This accelerated drug development and validation strategy has led to numerous clinical trials for the treatment of COVID-19 (Li and De Clercq, 2020; Liu et al., 2020). Despite several encouraging results, however, treatment of SARS-CoV-2 infection remains suboptimal and there is an urgent need to identify novel approaches to be used in clinical settings.

One of such approaches is to prevent the S protein/ACE2 interaction as a strategy to prevent SARS-CoV-2 entry into target cells. Several virtual screening campaigns have already identified small molecules able to bind residues at the interface between the RBD of SARS-CoV-2 S protein and the ACE2 receptor (Ghosh et al., 2020; Micholas and Jeremy, 2020; Senathilake et al., 2020; Utomo et al., 2020; Wang et al., 2020a; Yan et al., 2020; Zhou et al., 2020). In this paper, we have expanded on this area. Our results demonstrate that several potential binding sites exist in the SARS CoV-2 S protein and that the occupancy of these pockets reduces the ability of the S protein RBD to bind to the ACE2 consensus *in vitro*. Together, these results might help to define novel treatments by using SARS-CoV-2 entry inhibitors.

## Materials and Methods

### Virtual Screening

The electron microscopy (EM) model of SARS-CoV-2 Spike glycoprotein was downloaded from the Protein Data Bank (PDB ID: 6VSB). Missing loops were added from the Swiss-Model web-site (Wrapp et al., 2020). The obtained model was submitted to the Protein Preparation Wizard tool implemented into Maestro ver. 2019 (Schrödinger, 2019) to assign bond orders, adding all hydrogen atoms and adjusting disulfide bonds. The pocket search was performed by using the Fpocket website (Schmidtke et al., 2010).

The AutoDock4.2.6 suite (Morris et al., 2009) and the Raccoon2 graphical interface (Forli et al., 2016) were employed to carry out the virtual screening approach using the Lamarckian genetic algorithm (LGA). This hybrid algorithm combines two conformational research methods, the genetic algorithm and the local research. For the first low-accuracy screening, for each of the 2906 drugs, 3 poses were generated using 250,000 steps of genetic algorithm and 300 steps of local search, while in the second high-accuracy screening protocol, we generated 20 poses for each ligand, increasing the number of genetic algorithm steps to 25,000,000. The MGLTools were used to convert both ligands and each pocket into appropriate pdbqt files. Virtual screening was performed on a hybrid CPU/GPU HPC cluster equipped with 2 NVIDIA® Tesla® V100 GPUs and 560 Intel® Xeon® Gold and 64 AMD® EPYC® processors.

Each of the six selected RBD pockets were submitted to the AutoGrid4 tool, which calculates, for each bonding pocket, maps (or grids) of interaction, considering the different ligands and receptor-atom types through the definition of a cubic box. Subsequently, for each grid AutoDock4 calculates interaction energies (ADscore) that express the affinity of a given ligand for the receptor.

The library of FDA approved drugs has been obtained both from DrugBank (2106 compounds) (Drugbank, 2020) and from the Selleckchem website (FDA-approved Drug Library, 2020) (tot. 2638). Each database was converted to 3D and prepared with the LigPrep tool (Schrödinger, 2019) considering a protonation state at a physiological pH of 7.4. Subsequently, the two libraries were merged and deduplicated with Open Babel (O'Boyle et al., 2011), giving a total amount of 2,906 drugs. The bile acids (BA) focused library was prepared with the same protocol described above. All the images are rendered using UCSF Chimera (Pettersen et al., 2004).

### Molecular Dynamics (MD)

MD simulations were performed using the CUDA version of the AMBER18 suite (Lee et al., 2018) on NVIDIA Titan Xp and K20 GPUs, using the Amber ff14SB force field (Maier et al., 2015) to treat the protein. RBD was then immersed in a pre-equilibrated octahedral box of TIP3P water and the system was neutralized. The system was then minimized using energy gradient convergence criterion set to 0.01 kcal/mol Å^2^ in four steps involving: (i) an initial 5,000 minimization steps (2,500 with the steepest descent and 2,500 with the conjugate gradient) of only hydrogen atoms, (ii) 20,000 minimization steps (10,000 with the steepest descent and 10,000 with the conjugate gradient) of water and hydrogen atoms, keeping the solute restrained, (iii) 50,000 minimization steps (25,000 with the steepest descent and 25,000 with the conjugate gradient) of protein side chains, water and hydrogen atoms, (iv) 100,000 (50,000 with the steepest descent and 50,000 with the conjugate gradient) of complete minimization. Successively, the water, ions and protein side chains were thermally equilibrated in three steps: (i) 5 ns of NVT equilibration with the Langevin thermostat by gradually heating from 0K to 300K, while gradually rescaling solute restraints from a force constant of 10 to 1 kcal/mol Å^2^, (ii) 5 ns of NPT equilibration at 1 atm with the Berendsen thermostat, gradually rescaling restraints from 1.0 to 0.1 kcal/mol Å^2^, (ii) 5 ns of NPT equilibration with no restraints. Finally, a production run of 500 ns was performed using a timestep of 2 fs. The SHAKE algorithm was used for those bonds containing hydrogen atoms in conjunction with periodic boundary conditions at constant pressure and temperature, particle mesh Ewald for the treatment of long range electrostatic interactions, and a cutoff of 10 Å for nonbonded interactions.

### Dynamical Network Analysis

The Dynamical Network Analysis was performed on 500 ns long MD trajectories of the RBD domain using the plugin Carma ver. 0.8 (Glykos, 2006) implemented in VMD 1.9.2 (Humphrey et al., 1996), The optimal community distribution is calculated by using the Girvan–Newman algorithm (Girvan and Newman, 2002). Edges between each node (here defined as Cα atoms) were drawn between those nodes whose residues were within a default cut-off distance (4.5 Å) for at least 75% of our MD trajectories. Communities map analysis and representation were obtained using the NetworkView tool, implemented in VMD 1.9.2.

### Chemistry

OCA, BAR704, BAR501, and BAR502 were synthesized as previously described (Festa et al., 2014; Sepe et al., 2016).

### ACE2/SARS-CoV-2 Spike Inhibitor Screening Assay Kit

We tested the selected compounds (UDCA, T-UDCA, G-UDCA, CDCA, G-CDCA, OCA, BAR501, BAR502, BAR704, betulinic acid, oleanolic acid, glycyrrhetinic acid, potassium canrenoate) using the ACE2: SARS-CoV-2 Spike Inhibitor Screening Assay Kit (BPS Bioscience Cat. number #79936) according to the manufacturer's instructions. All compounds were tested at different concentrations in a range from 0.01 to 100 μM. In addition, a concentration-response curve for the Spike protein (0.1–100 nM) was constructed to confirm a concentration-dependent increase in luminescence. A spike concentration of 5 nM was used for the screening of the compounds. Briefly, thaw ACE2 protein on ice and dilute to 1 μg/ml in PBS. Use 50 μL of ACE solution to coat a 96-well nickel-coated plate and incubate 1 h at room temperature with slow shaking. Wash the plate 3 times and incubate for 10 min with a Blocking Buffer. Next, add 10 μL of inhibitor solution containing the selected compound and incubate for 1 h at room temperature with slow shaking. For the “Positive Control” and “Blank,” add 10 μL of inhibitor buffer (5% DMSO solution). After the incubation, thaw SARS-CoV-2 Spike (RBD)-Fc on ice and dilute to 0.25 ng/μL (~5 nM) in Assay Buffer 1. Add the diluted Spike protein to each well, except to the blank. Incubate the reaction for 1 h at room temperature, with slow shaking. After 3 washes and incubation with a Blocking Buffer (10 min), treat the plate with an Anti-mouse-Fc-HRP and incubate for 1 h at room temperature with slow shaking. Finally, add an HRP substrate to the plate to produce chemiluminescence, which then can be measured using FluoStar Omega microplate reader.

In another experimental setting, we have tested the selected compounds using the ACE2: SARS-CoV-2 Spike Inhibitor Screening Assay Kit with a slight modification to the protocol. In particular, tested compounds were pre-incubated for 2 h with the Spike-RBD, and immediately afterwards the mix was incubated with ACE2 coated on the 96-well plate.

### Quantitative Analysis of the Anti-SARS-CoV-2 IgG Antibodies

To confirm the validity of the assay used in this study, five remnants of plasma samples used to test levels of anti-SARS CoV2 IgG in post COVID-19 patients were used. The original samples were collected at the blood bank of Azienda Ospedaliera of Perugia from post COVID-19 donors who participate to a program of plasma biobanking. An informed and written consent was signed by donors recruited in this program. The program's protocol included the quantitative analysis of the anti-SARS-CoV-2 IgG antibodies directed against the subunits (S1) and (S2) of the virus spike protein. IgGs were therefore measured by chemiluminescence immunoassay (CLIA) technology (LIAISON®SARS-CoV-2 IgG kit, DiaSorin®, Saluggia, Italy). Leftovers of five samples from this assay of ≈ 40–50 μL whose destiny was to be discharged were used to validate the SARS-CoV-2/ACE2 assay used in our study. While donors have provided a written informed consent for plasma donation as mentioned above, and no blood samples were taken specifically for this study, we (SB and DF) have contacted the five donors whose serum leftovers were used in this study by a phone call and asked the permission to use the sample remnants. The permission was granted by all five donors. We wish to thank all of them for the kind collaboration.

## Results

### Virtual Screening of the FDA-Approved Drug Library

**Figure 1:**
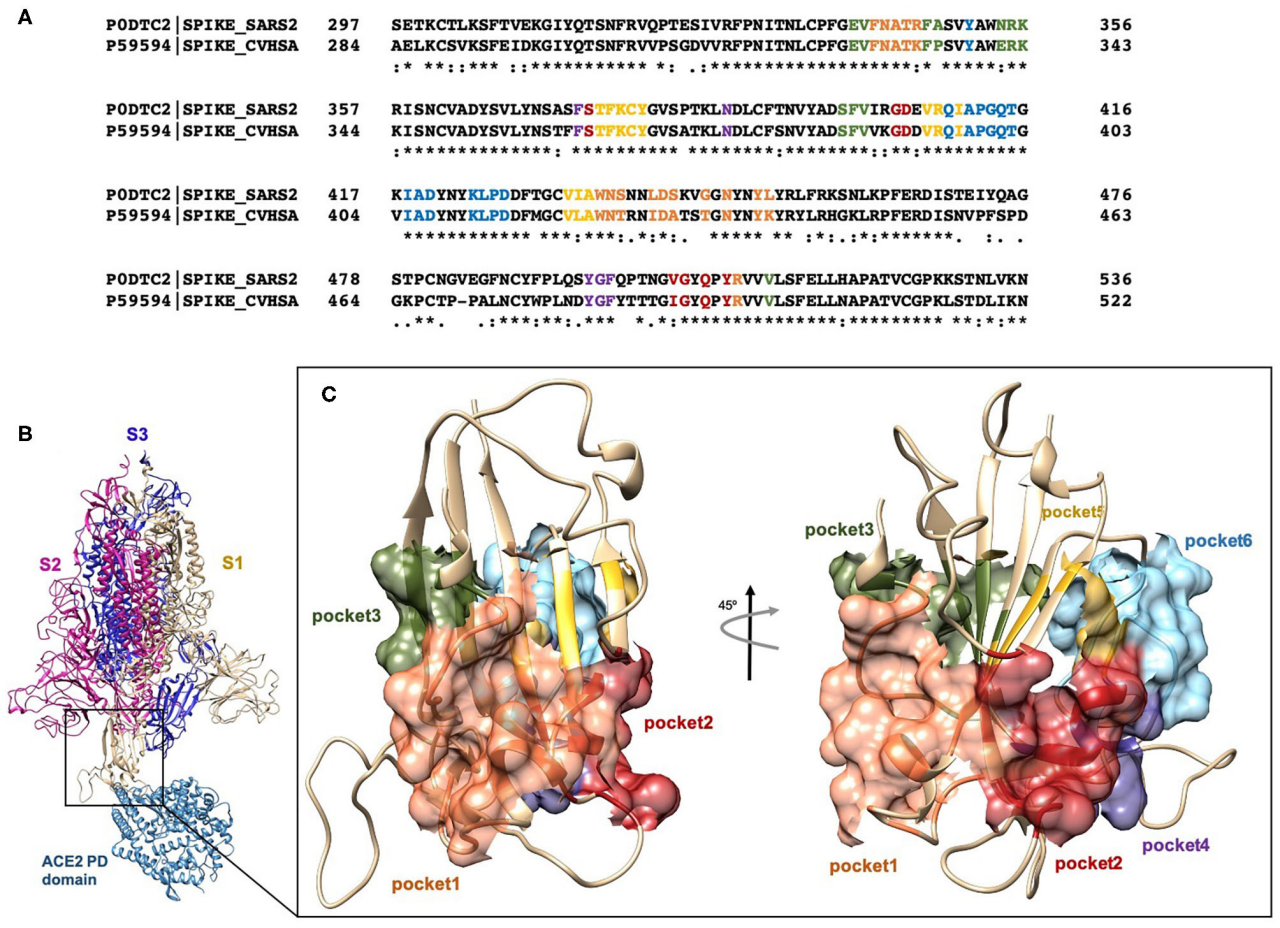
**(A)** Clustal Omega alignment of RBD regions of SARS-CoV and SARS-CoV-2 Spike protein. Residues bearing to different pockets are colored respectively yellow (Pocket 1), green (Pocket 2), light blue (Pocket 3), magenta (Pocket 4), red (pocket 5), and dark slate blue (Pocket 6). **(B)** Cartoon representation of the trimer of SARS-2 Spike protein in complex with the PD domain of ACE2. Complex obtained through the superposition of the PDB structures 6VSB and 6M0J. **(C)** Surface representation of the six selected pockets used for the screening.

With the aim to identify chemical scaffolds capable of inhibiting ACE2/Spike interaction by targeting the RBD of the S1 domain of the SARS-CoV-2 (Figure 1B), we carried out a virtual screening campaign on an FDA-approved drug library, using the RBD 3D structure obtained from the Protein Data Bank (PDB ID 6SVB; Chain A, residues N331-A520) (Wrapp et al., 2020). Missing regions in the structure were built through the SwissModel webserver (Bertoni et al., 2017). A pocket search was performed with the Fpocket web-server (Le Guilloux et al., 2009), resulting in the identification of ≈ 300 putative pockets on the whole trimeric structure of the S protein. This search was further refined to identify selected pockets in the RBD according to three main factors: (i) the potential druggability, i.e., the possibility of interfering, directly or through an allosteric mechanism, with the interaction with ACE2; (ii) the flexibility degree of the pockets, i.e., excluding pockets defined, even partially, by highly flexible loops, whose coordinates were not defined in the experimental structure; (iii) sequence conservation with respect to SARS-CoV RBD (Figure 1A). On these bases, 6 pockets were selected on the RBD and numbered according to the Fpocket ranking (Figures 1A,C).

First, these pockets were used for the virtual screening of 2,906 FDA-approved drugs from the DrugBank and the Selleckchem websites, using the AutoDock4.2.6 program (Morris et al., 2009) and the Raccoon2 graphical user interface (Forli et al., 2016). This step was followed by a high-accuracy screening, based on the binding affinity predicted by AutoDock4 (ADscore), with a focus on the results showing an ADscore lower than −6 kcal/mol.

These studies allowed the identification of several compounds with steroidal and triterpenoid scaffold, including glycyrrhetinic acid, betulinic acid and the corresponding alcohol (betulin), canrenone and the corresponding open form on the γ-lactone ring as potassium salt (potassium canrenoate), spironolactone and oleanolic acid, showing robust binding selectivity toward the RBD's pocket 1 (Table 1).

**Table 1:** Results of the screening of FDA approved drugs on the RBD region of the Spike protein of SARS-CoV-2 with the Autodock 4.2.6 program.

**Compound**	**ADscore**	**Pocket**
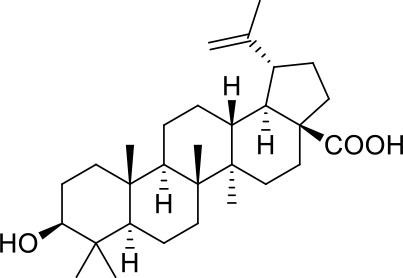	−8.1	1
Betulinic Acid		
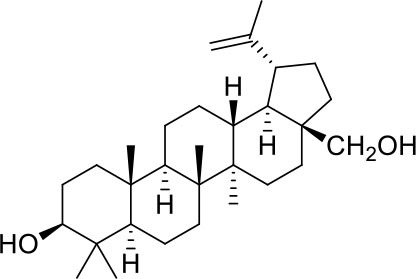	−7.4	1
Betulin		
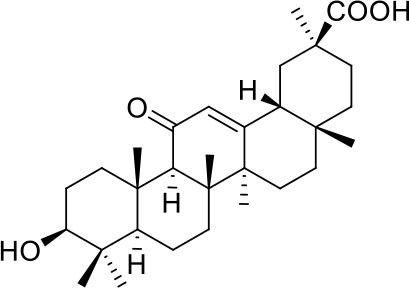	−8.6	1
Glycyrrhetinic acid		
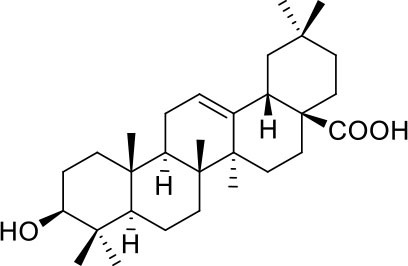	−8.2	1
Oleanolic acid		
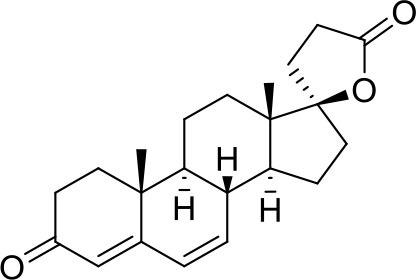	−7.9	1
Canrenone		
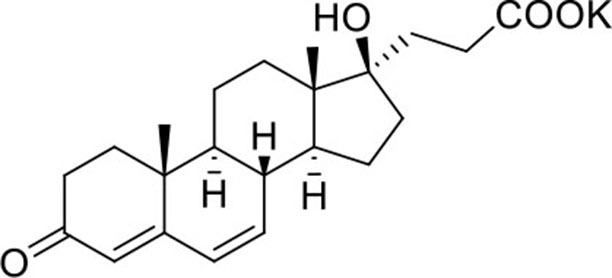	−6.9	1
Potassium Canrenoate		
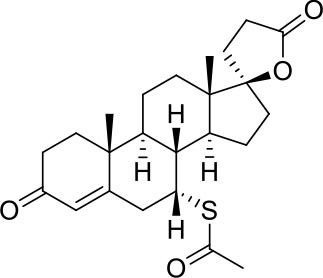	−6.2	1
Spironolactone		

*Binding affinity values (ADscore) are expressed in kcal/mol*.

Pocket 1, located on the β-sheet in the central core of the RBD, is the less conserved among the screened, presenting five conservative (R346K, S438T, L440I, S442A) and two non-conservative (G445T and L451K) mutations from SARS-CoV-2 to SARS-CoV.

**Figure 2:**
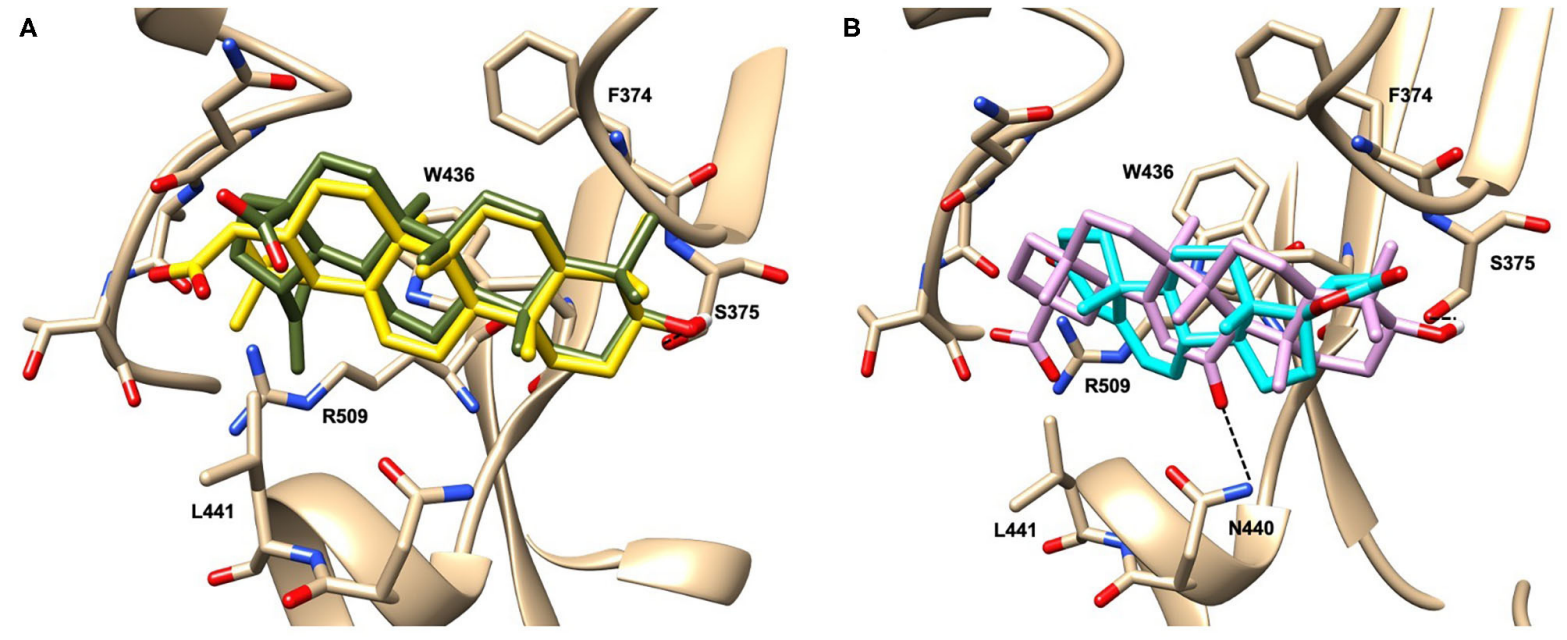
Graphical representation of the binding mode of the best compounds resulting from the screening in pocket 1. The RBD region is represented in transparent surface colored by residues hydrophobicity. Color codes are: dodger blue for the most hydrophilic regions, white, to orange-red for the most hydrophobic. **(A)** Betulinic acid (dark olive-green stick) and oleanolic acid (gold stick). **(B)** Glycyrrhetinic acid (plum stick) and potassium canrenoate (cyan stick). For clarity reasons hydrogen atoms are omitted and only interacting aminoacids are displayed in sticks.

Glycyrrhetinic acid, the best compound according to the AD score, binds the pocket through both hydrophobic and polar interactions. The triterpenoid scaffold relied between the hydrophobic side of the β-sheet core of RBD, defined by W436, F374 and the side chain of R509, and L441 on the other side, engaging hydrophobic contacts. In addition, the binding is reinforced by ionic contacts between the carboxyl group with R509, and by hydrogen bonds between the carbonyl group with N440 and the hydroxyl group with S375. Oleanolic acid and betulinic acid showed similar binding modes with the main difference in the carboxylic groups oriented toward the solvent. Finally, potassium canrenoate showed a different orientation of the steroidal system within the binding site, with the carboxylic function weakly bonded to S375 (3.1 Å), and the π-system of rings A and B stacked between W436 and L441 (Figure 2).

Because the above mentioned triterpenoids have been identified as natural ligands for two bile acid activated receptors, the Farnesoid-X-Receptor (FXR) and G protein Bile Acid Receptor (GPBAR)-1 (Sepe et al., 2015; De Marino et al., 2019; Fiorucci and Distrutti, 2019), we have further investigated whether mammalian ligands of these receptors were also endowed with the ability to bind the above mentioned RBD's pockets. More specifically, oleanolic, betulinic and ursolic acids have been proved to act as selective and potent GPBAR1 agonists (Sato et al., 2007; Genet et al., 2010; Lo et al., 2016), while glycyrrhetinic acid, the major metabolic component of licorice, and its corresponding saponin, glycyrrhizic acid, have been shown to act as dual FXR and GPBAR1 agonists in transactivation assay (Distrutti et al., 2015), also promoting GLP-1 secretion in type 1-like diabetic rats (Wang et al., 2017).

Bile acids are steroidal molecules generated in the liver from cholesterol breakdown (Fiorucci and Distrutti, 2019). Primary bile acids include cholic acid (CA) and chenodeoxycholic acid (CDCA), which have been recognized as functioning as the main FXR ligands in humans (Fiorucci and Distrutti, 2019). Secondary bile acids, deoxycholic acid and lithocholic acid (DCA and LCA) generated by intestinal microbiota, are preferential ligands for GPBAR1 (Maruyama et al., 2002; Fiorucci and Distrutti, 2019). Ursodeoxycholic acid (UDCA), which is a primary bile acid in mice, but a “tertiary” bile acid found in trace in humans, is, along with CDCA, the only bile acid approved for clinical use, and is a weak agonist for GPBAR1 and considered a neutral or weak antagonist toward FXR (Carino et al., 2019).

Taking into account the structural similarity and the ability to bind the same receptor systems, we have carried out an in-depth docking analysis of natural bile acids and their semisynthetic derivatives currently available in therapy or under pre-clinical and clinical development (De Marino et al., 2019) and tested them for their ability to bind the above-mentioned pockets in the RBDs of SARS-CoV-2 S protein (Table 2).

**Table 2:** Results of the screening of natural bile acids on the RBD region of the Spike protein of SARS-CoV-2 with the Autodock 4.2.6 program.

**Compound**	**ADscore**	**Pocket**
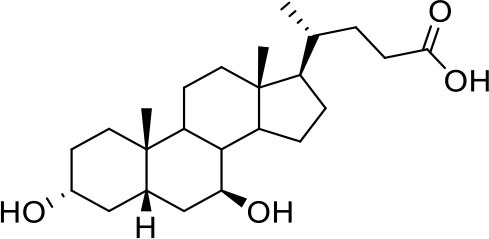	−7.0	5
Ursodeoxycholic acid (UDCA)		
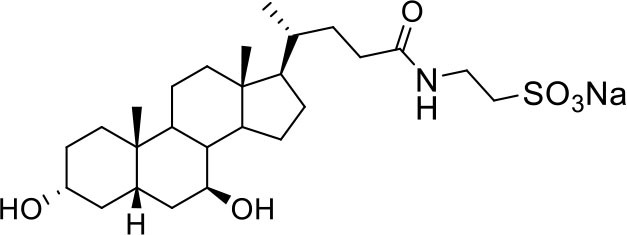	−7.0	5
Tauro-ursodeoxycholic Acid (T-UDCA)		
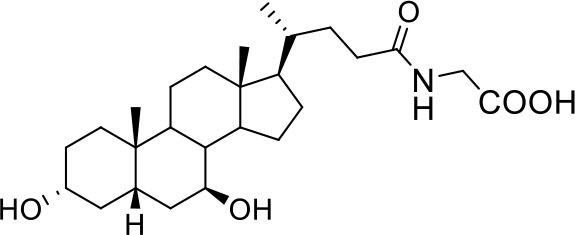	−7.3	5
Glyco-ursodeoxycholic Acid (G-UDCA)		
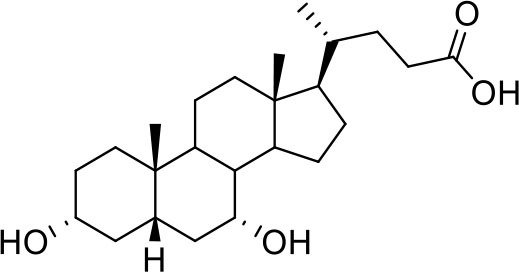	−7.3	5
Chenodeoxycholic acid (CDCA)		
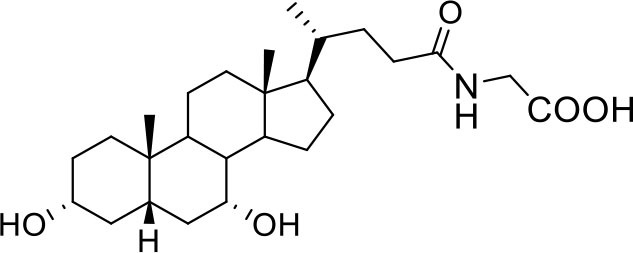	−7.6	5
Glyco-chenodeoxycholic acid (G-CDCA)		
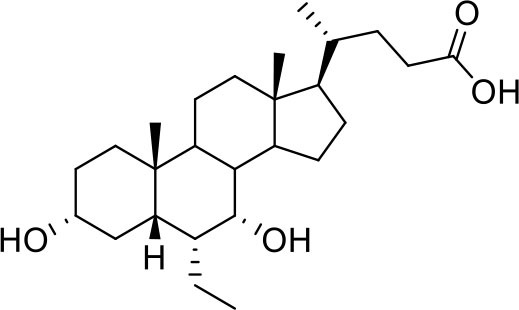	−7.6	5
Obeticholic acid (OCA)		
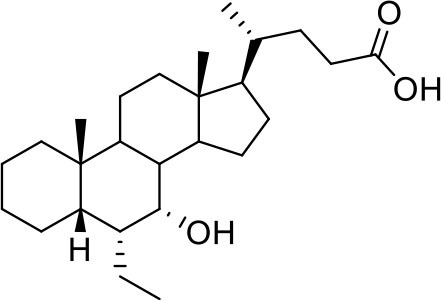	−7.2	5
BAR704		
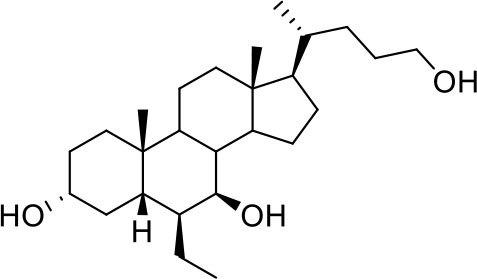	−6.9	5
BAR501		
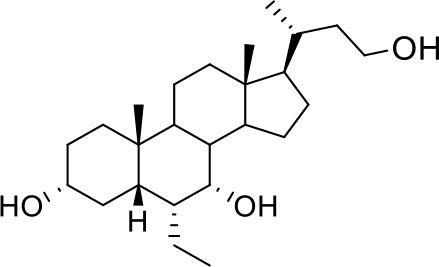	−7.3	5
BAR502		

*Binding affinity values (ADscore) are expressed in kcal/mol*.

**Figure 3:**
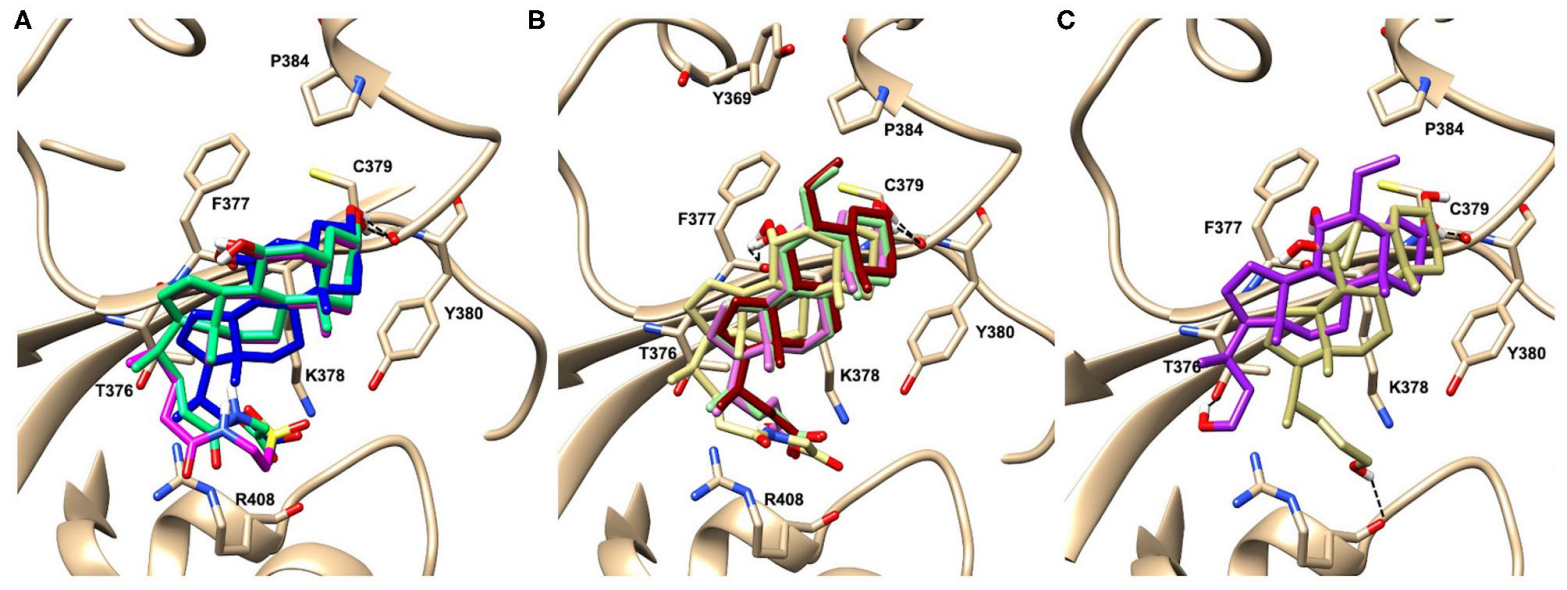
Graphical representation of the binding mode of the best compounds resulting from the screening in pocket 5. The RBD region is represented in tan cartoon, while the pocket 5 residues as transparent surface colored by residues hydrophobicity. Color codes are: dodger blue for the most hydrophilic regions, white, to orange-red for the most hydrophobic. **(A)** UDCA (blue stick), T-UDCA (magenta stick) and G-UDCA (spring-green stick); **(B)** CDCA (orchid stick), OCA (light-green stick), BAR704 (dark-red stick) and G-CDCA (khaki stick); **(C)** BAR501 (gold stick) and BAR502 (purple stick). For clarity reasons hydrogen atoms are omitted and only interacting aminoacids are displayed in sticks.

As shown in Table 2, natural bile acids and their semi-synthetic derivatives exhibit higher affinity scores for pocket 5. This pocket (Figures 3A–C) included residues bearing to the central β-sheet core but on a different side than pocket 1. The pocket resulted to be very conserved, showing only one mutation, I434L, from SARS-CoV-2 to SARS-CoV.

In the binding mode of UDCA, the carboxylic group on the side chain is positioned between K378 and R408 and the steroidal scaffold is placed in a hydrophobic surface defined by the side chains of K378, T376, F377, Y380 and P384. Additionally, the 3β-hydroxyl group on ring A forms H-bonds with the backbone carbonyl of C379. The corresponding glycine and taurine-conjugated derivatives (G-UDCA and T-UDCA, respectively) showed the same ionic interactions of their negatively charged groups with K378 and R408. Albeit the greater length of the side chain, the H-bond with the backbone carbonyl of C379 induces a shift of the steroidal system toward T376, and an additional π-interaction between the electron density of the glycine amide region and the guanidine moiety of R408. This results in a better score for G-UDCA, and a reduction in the case of T-UDCA, likely due to a non-optimal arrangement of the taurine moiety within the binding pocket. CDCA showed a very similar binding mode, with the only difference that it formed an additional H-bond with the backbone carbonyl of F377 due to the modification in the configuration of the C-7 hydroxyl group (α-oriented in CDCA and β-oriented in UDCA). As for G-UDCA, also G-CDCA established the same H-bonds network of the parent CDCA, while the steroidal core slightly shifted as described for G-UDCA. Interestingly, AD scores of G-UDCA and G-CDCA clearly indicated that the H-bond between the hydroxyl group at C-7 and F377 does not contribute significantly to the binding mode.

With respect to CDCA, the introduction of the ethyl group at the C-6 position as in OCA and in BAR704 improves the internal energy of the ligand (−0.27 for CDCA vs. −0.59 and −0.60 kcal/mol for OCA and BAR704, respectively) and further favors the binding (Figure 3B), even if, albeit in close proximity of P384 and Y369, the 6-ethyl group did not show any particular contact within the RBD region.

BAR501, a neutral UDCA derivative, with an alcoholic side-chain end group and the ethyl group at C-6 β-oriented showed a very similar binding mode compared to the parent compound, with the side chain hydroxyl group H-bonded to R408. Finally, BAR502, with a one carbon less on the side chain positioned the steroidal core as for G-CDCA, thus allowing the C-23 OH group H-bonding with the side chain hydroxyl group of T376.

### Dynamical Network Analysis

**Figure 4:**
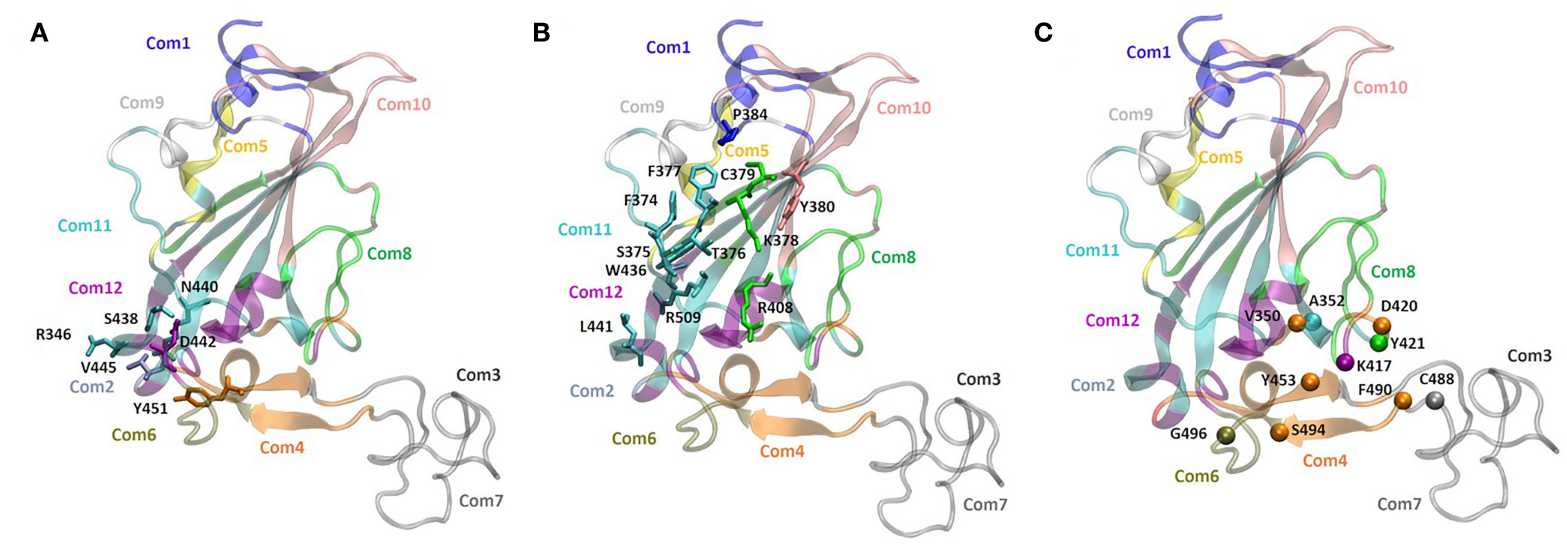
Community network representation of the RBD domain and community residue members of **(A)** pocket 1 (N440, S438, R346, D442, V445, and Y451), **(B)** pocket 5 (Y380, K378, F377, R408, C379, T376, P384, F374, S375, W436, L441, and R509). **(C)** Highest score edge connectivity residues retrieved on the basis of the betweenness matrix. Spheres indicate the Cα atoms of residues that occur in a majority of shortest paths connecting nodes in different communities.

To support our hypothesis about the allosteric inhibitory potential of the identified pockets, we performed a dynamical network and community map analysis on 500 ns of molecular dynamics (MD) simulations of the RBD domain. Overall, the network analysis found 12 communities (Com1-Com12) (Figures 4A–C and Table 3). Each community corresponds to a set of residues in the RBD domain that move in concert with each other. By definition, nodes (defined here as the Cα atoms) belonging to the same community are highly interconnected, however, few nodes (called “critical”) may also connect to the edge of different communities by a metric called betweenness (Figure 4C). In our network analysis, the 12 communities identified are distributed as follows: the RBM region resulted in a split into three communities (Com4, Com6, and Com7), with Com4 including the short β-sheet, while Com6 and Com7 include residues of the binding loops G496-Y505 and F456-F490 (Table 3), respectively. Pocket 1 and pocket 5 residues lie mainly in Com11 (Table 3), but few residues are included in other communities, in particular pocket 1 residue Y451 in Com4 and residues S438 and D442 in Com12, while pocket 5 residues T376, K378, C379, R408 in Com8 and Y380 Com10. In order to highlight the potential allosteric communication among the different communities, we analyzed the edge betweenness (Figure 4C), which is a measure of the shortest paths between pairs of nodes belonging to two different communities. We found that communities including residues of pocket 1 and pocket 5 indirectly communicate with Com6 and Com7, through Com4. In particular, Com8, Com10, Com11, and Com12, including most of the residues in both pockets 1 and 5, were connected to Com4, which in turn was strongly connected to Com6 and weakly to Com7, thus indicating at least a strong potential allosteric communication among the pockets and the loops at the receptor interface.

**Table 3:** Community map distribution of the RBD domain, retrieved after 500 ns-long MD simulation.

**Community**	**N. of members**	**Residues**	**Color code cartoon**
Com1	14	N334; C361; V382; P384; T385; L387; D389; V524-K529;	Blue
Com2	1	V445	Ice-blue
Com3	1	G476	Dark-gray
Com4	18	V350; G416; D420; G446-R454; F456; F490-S494;	Orange
Com5	7	C336; E340; F342; A344	Yellow
Com6	9	Y495-G502; G504	Tan
Com7	33	L455;R457-A475; S477-Y489	Light-Gray
Com8	20	T376; K378; C379; R408; I410-T415; I418; A419; Y421; Y423-P426; D428; T430; V511	Green
Com9	9	A363-Y369; S371; S383	White
Com10	30	L335; R355-N360; V362; Y380; G381; K386; L390-V395; D427-F429; L513-T523	Pink
Com11	38	V341; N343;T345; R346; Y351-N354; N370; A372-S375; F377; Y396-F400; N422; G431-A435; N437; N439-L441; S443; P507-V510; V512	Cyan
Com12	16	V401-V407; Q409; K417; W436; S438; D442; K444; V503; Y505; Q506	Purple

### *In vitro* Screening

**Figure 5:**
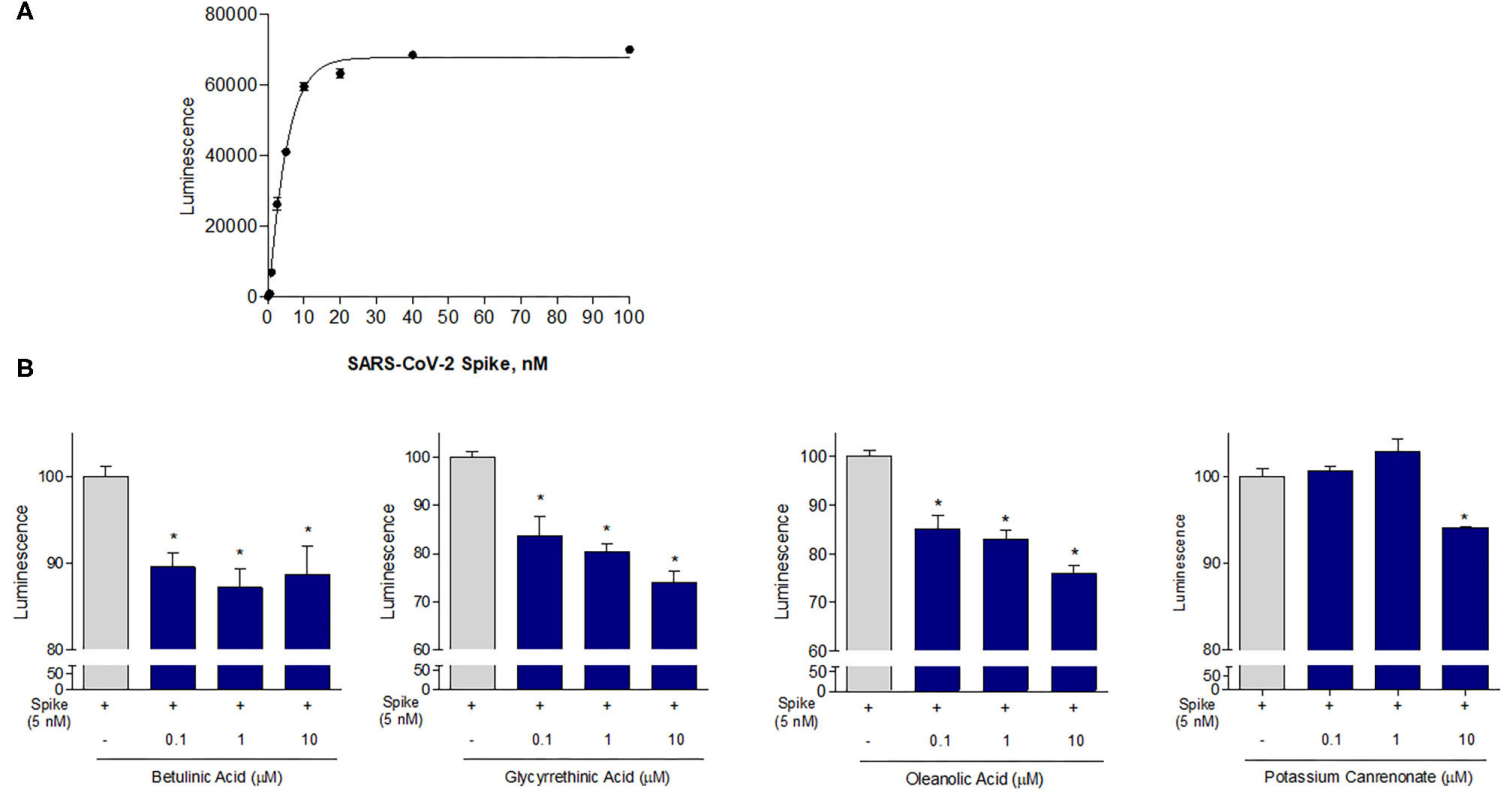
The ACE2:SARS-CoV-2 Spike Inhibitor Screening assay was performed as described in Material and Method section. Data shown are: **(A)** SARS-CoV-2 Spike binding to immobilized ACE2, using an increasing dose of Spike protein (0, 5–100 nM); Luminescence was measured using a Fluo-Star Omega fluorescent microplate reader. **(B)** Betulinc acid, glycyrrethinic acid, oleanolic acid and potassium canrenonate were tested at different concentration (0.1, 1, and 10 μM), to evaluate their ability to inhibit the binding of Spike protein (5 nM) to immobilized ACE2, by using the ACE2:SARS-CoV-2 Spike Inhibitor Screening assay Kit. Luminescence was measured using a Fluo-Star Omega fluorescent microplate reader. Luminescence values of Spike 5 nM were arbitrarily set to 100%. Results are expressed as mean ± standard error. *p < 0.05 vs. Spike 5 nM. Data are the mean ± SE, *n* = 3.

Given the results of the virtual screening, we have then investigated whether the agents mentioned in Tables 1, 2 impact on the binding of S protein to the ACE2 receptor. For this purpose, a Spike/ACE2 Inhibitor Screening Assay Kit was used. The assay is designed for screening and profiling inhibitors for RBD/ACE2 interaction. To validate the assay, we first performed a concentration-response curve by adding increasing concentrations of the Spike RBD (0.1–100 nM) and confirmed a concentration-dependent increase of luminescence (*n* = 5 experiments, Figure 5A). Since the curve was linear in the range from 0.1 to 10 nM, we have used the concentration of 5 nM for all the following assays. As illustrated in Figure 5, we found that incubating the Spike RBD with betulinic acid, glycyrrhetinic acid, oleanolic acid, and potassium canrenoate (the active metabolite of spironolactone) results in concentration-dependent reductions of the binding of S Spike RBD to the ACE2 receptor. While all agents effectively reversed the binding at a concentration of 10 μM, betulinic acid and oleanolic acid showed a significant inhibition at a concentration of 0.1 and 1 μM, respectively (*n* = 3 replicates).

**Figure 6:**
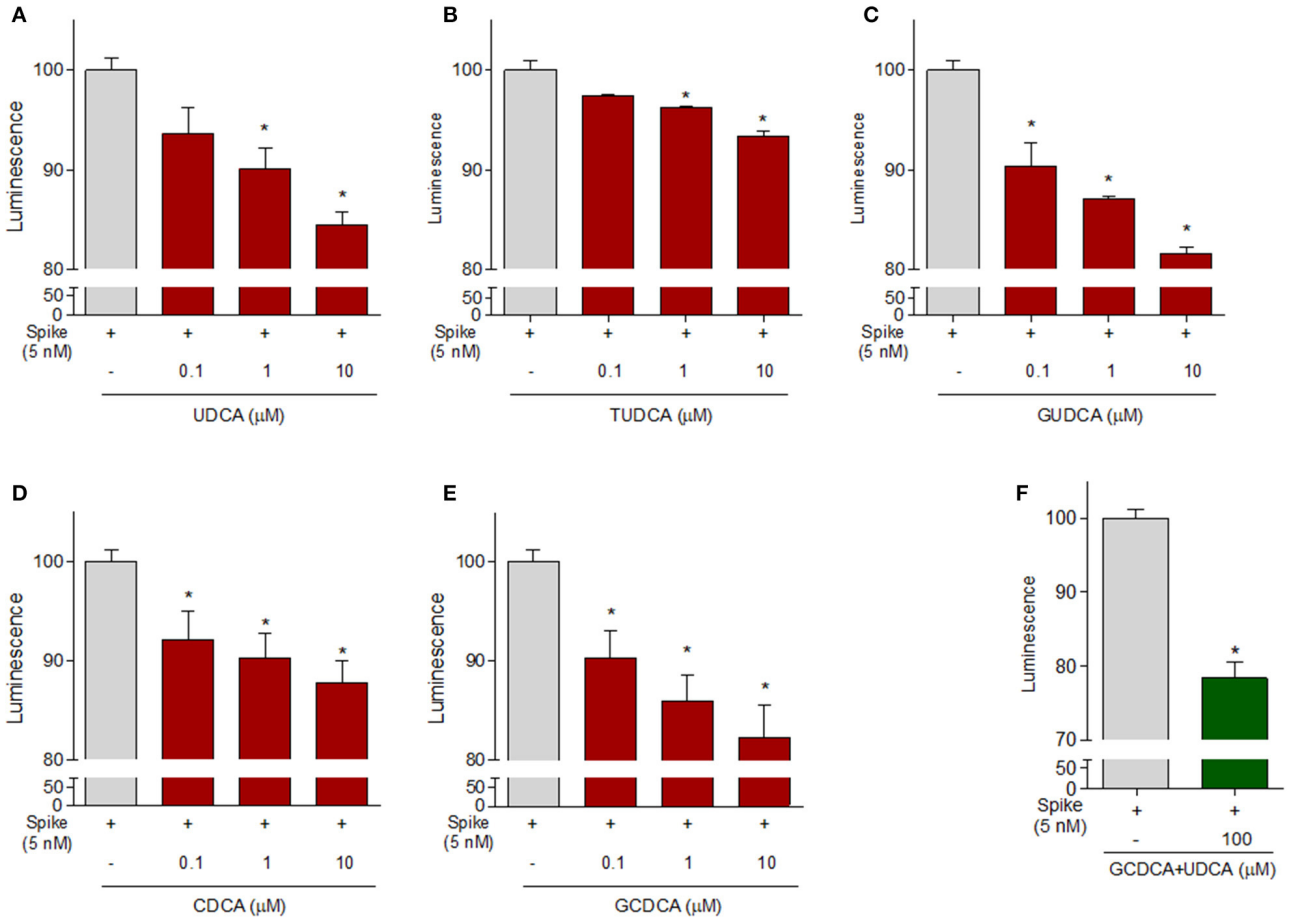
The ACE2:SARS-CoV-2 Spike Inhibitor Screening assay was performed as described in Material ad Method section. Natural bile acids (A) UDCA, (B) TUDCA, (C) GUDCA, (D) CDCA, (E) GCDCA (0.1, 1 and 10μM) and (F) a combination of GCDCA + UDCA (100μM), were tested to evaluate their ability to inhibit the binding of Spike protein (5 nM) to immobilized ACE2, by using the ACE2:SARS-CoV-2 Spike Inhibitor Screening assay Kit. Luminescence was measured using a Fluo-Star Omega fluorescent microplate reader. Luminescence values of Spike 5 nM were arbitrarily set to 100%. Results are expressed as mean ± standard error. *p < 0.05 vs. Spike 5 nM. Data are the mean ± SE, *n* = 3.

Because these data demonstrate that betulinic acid and oleanolic acid were effective in inhibiting the binding of the S protein RBD to ACE2, and the two triterpenoids were known for their ability to modulate GPBAR1, we then tested whether natural GPBAR1 bile acids ligands were also effective in reducing the SARS-CoV-2-ACE2 interaction. As illustrated in Figure 6, the secondary bile acid UDCA and its taurine conjugate, T-UDCA, caused a slight and dose dependent inhibition of the bind of the S protein RBD to the ACE2 receptor (Figures 6A,B). G-UDCA, i.e., the main metabolite of UDCA in humans, inhibits the RBD binding to the ACE2 receptor by ~20% in a concentration dependent manner. Similar concentration dependent effects were observed with CDCA and to a greater extent with its metabolite, G-CDCA (Figure 6D). A combination of UDCA and G-CDCA exerted a slight additive effect, confirming that UDCA itself has a very limited inhibitory activity.

**Figure 7:**
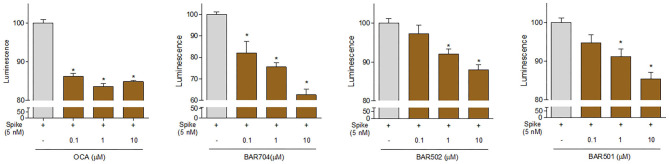
The ACE2:SARS-CoV-2 Spike Inhibitor Screening assay was performed as described in Materials and Methods section. The semi-synthetic bile acid receptor agonists OCA, BAR704, BAR502, and BAR501, were tested at different concentration (0.1, 1, and 10 μM) to evaluate their ability to inhibit the binding of Spike protein (5 nM) to immobilized ACE2, by using the ACE2:SARS-CoV-2 Spike Inhibitor Screening assay Kit. Luminescence was measured using a Fluo-Star Omega fluorescent microplate reader. Luminescence values of Spike 5 nM were arbitrarily set to 100%. Results are expressed as mean ± standard error. *p < 0.05 vs. Spike 5 nM. Data are the mean ± SE, *n* = 3.

Continuing the *in vitro* screening, we investigated whether the semisynthetic bile acid derivatives obeticholic acid (OCA), BAR704, BAR501, and BAR502, exerted comparable or better effects than G-CDCA. As illustrated in Figure 7, adding OCA to the incubation mixture reduced the binding of SARS-CoV-2 S spike to ACE2 by ≈20%. In contrast, BAR704, a 3-deoxy 6-ethyl derivative of CDCA, and a highly selective and potent FXR agonist, was significantly more effective and reduced the binding by ~40% at the dose of 10 μM. On the other hand, BAR501 and BAR502, alcoholic derivatives of UDCA and CDCA, respectively, were only slightly effective in reducing the binding of S protein RBD to ACE2.

**Figure 8:**
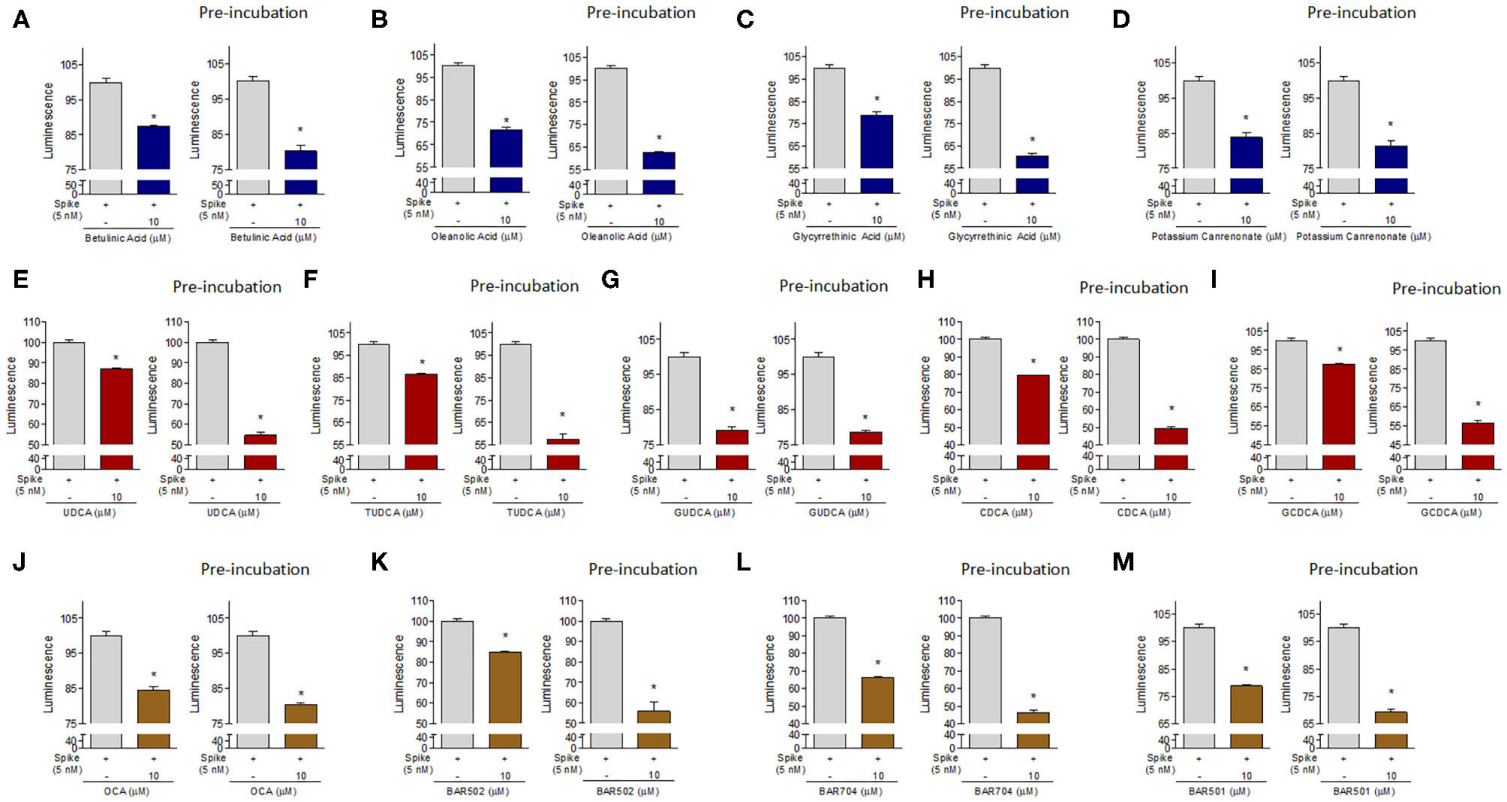
The ACE2:SARS-CoV-2 Spike Inhibitor Screening assay was performed as described in Material ad Method section. The selected compounds were tested at 10 μM to evaluate their ability to inhibit the binding of Spike protein (5 nM) to immobilized ACE2, according the ACE2:SARS-CoV-2 Spike Inhibitor Screening assay Kit instructions or with a modified protocol in which we have performed a pre-incubation of these compounds with Spike-RBD (2 h). Tested compounds were: **(A)** Betulinic Acid, **(B)** Oleanolic Acid, **(C)** Glycyrrethinic Acid, **(D)** Potassium Canrenoate, **(E)** UDCA, **(F)** TUDCA, **(G)** GUDCA, **(H)** CDCA, **(I)** GCDCA, **(J)** OCA, **(K)** BAR502, **(L)** BAR704, **(M)** BAR501. Luminescence was measured using a Fluo-Star Omega fluorescent microplate reader. Luminescence values of Spike 5 nM was arbitrarly setted to 100%. Results are expressed as mean ± standard error. *p < 0.05 vs. Spike 5 nM. Data are the mean ± SE, *n* = 3.

To further confirm our results, additional *in vitro* experiments were carried by pre-incubating the Spike RBD alone with 10 μM of selected compound. As shown in Figure 8, several of the compounds exhibited a greater ability to reduce the interaction between Spike and ACE2 when pre-incubated with Spike-RBD compared with the standard incubation performed in the same experiment (Figures 8A–M, ^*^p < 0.05). In particular, we found that oleanolic and glycyrrhetinic acid reduced the binding of Spike-RBD to ACE2 by 40% when pre-incubated with the RBD, whereas betulinic acid and potassium canrenoate showed no additional gain (Figures 8A–D, ^*^p < 0.05). Several natural bile acids, such as UDCA, T-UDCA, CDCA and G-CDCA, exerted a greater inhibitory effect when preincubated with Spike reaching ~45–50% of binding inhibition (Figures 8E–I, ^*^p < 0.05). Among the semisynthetic bile acid derivatives, their pre-incubation with Spike-RBD improved the efficacy of OCA (40%) and BAR502 (45%) (Figures 8J,K, ^*^p < 0.05) and BAR704 that reduced the interaction ACE2/Spike-RBD by 55% (Figure 8L, ^*^p < 0.05). These results suggested that the reduction of Spike-ACE2 interaction is actually due to the binding of tested compounds with the residues of Spike-RBD, thus confirming the molecular docking results.

### Effects of Plasma Samples From Post-COVID-19 Convalescent Patients on Spike RBD –ACE2 Interaction

To confirm the concept that binding the pockets in the central β-sheet core of Spike RBD effectively prevents its interaction with the consensus of ACE2 receptor, we then carried out a set of control experiments using remnants of the plasma samples from five donors that have recovered from COVID-19. These donors had a slightly different title of anti SARS-CoV-2 antibodies (See Material and Methods, Table 4), but all the dilutions tested effectively inhibited the Spike RBD binding to ACE2 in our assay system by more than 95%. These data highlight that the test used in this paper correctly identify the binding of SARS-CoV-2 RBD to ACE2, but the levels of inhibition, were, as expected, significantly lower than those that could be reached by anti-SARS-CoV-2 antibodies.

**Table 4:** Percentage of inhibition of the Spike:ACE2 binding.

		**% of Binding Inhibition**		
**Patient ID**	**Antibody Title**	**5 μL of Serum**	**10 μL of Serum**	**20 μL of Serum**
1	96.6 AU/mL	98.6	99.5	99.6
2	170 AU/mL	99.3	99.4	99.3
3	89.4 AU/mL	98.1	99.3	99.4
4	125 AU/mL	98.8	99.3	99.4
5	146 AU/mL	95.7	96.9	97.3

*Serum efficacy has been calculated in ACE2:SARS-CoV-2 Spike Inhibitor Screening Assay Kit as percent of inhibition of Spike RBD binding to ACE2 binding obtained using SPIKE at 5 nM, arbitrarily set as 100%*.

## Discussion

In this study we report the results of a virtual screening campaign designed to identify natural and clinically available compounds that might have utility in the prevention/treatment of the SARS-CoV-2 infection. In the light of the need of effective therapies to be rapidly tested for preventing or treating COVID-19, we initiated an *in silico* campaign to identify putative molecular targets that could be exploited to prevent the interaction of the SARS-CoV-2 Spike protein with the cellular machinery hijacked by the virus to enter target cells. To this end, we identified the Spike RBD as a potential pharmacological target. Accordingly, we developed the concept that putative pockets on the surface of the central β-sheet core of the S protein RBD could be exploited eventually to prevent the binding of the virus to ACE2.

Our *in silico* screening has allowed the identification of six potentially druggable pockets and the virtual screening of the FDA-approved drug library identified steroidal compounds as potential hits against two pockets, namely pocket 1 and pocket 5. Interestingly, high accuracy docking demonstrated that flat steroidal scaffolds (i.e., A/B rings junction *in trans* configuration Table 1) prefer pocket 1, while compounds with the A/B junction in *cis* configuration (Table 2, such as bile acids) show greater affinity for pocket 5.

Our *in vitro* testing has largely confirmed the functional relevance of the two main pockets identified by *in silico* analyses. One important finding of this study has been that several steroidal molecules were effective inhibitors of the binding of the RBD to ACE2 *in vitro*. In particular, the most interesting compounds in Table 1, glycyrrhetinic and oleanolic acid, showed good agreements in terms of docking AD score and in their ability to inhibit the spike/ACE2 interaction *in vitro*. The results also suggested that the main determinant for the inhibition efficacy is the hydrophobicity, as demonstrated by oleanolic acid, lacking any charge interaction within the pocket and resulting the most effective inhibitor in the series.

Hydrophobicity is also the main determinant of the activity of the bile acids and their semisynthetic derivatives, as demonstrated by CDCA, the corresponding glyco-conjugated derivative (G-CDCA) and its semisynthetic derivatives OCA, BAR704, and BAR502. Indeed, comparing the binding mode and the inhibition efficacy of CDCA and OCA with the related 6-ethyl derivative BAR704 highlighted the critical effect of the 6α-ethyl group in the inhibition activity and the negligible contribution of the 3β-hydroxyl group. The above positive effect could be explained considering the internal energy contribution of these ligands to the AD score, as well as the possibility of engaging more hydrophobic contacts. Indeed, the AD score internal energy contribution, significantly higher for the 6-ethyl derivatives, represents a measure of the conformational energy of the bound *vs*. unbound state of the ligand, thus indicating that the ethyl group facilitates the assumption of the bioactive conformation. Moreover, the analysis of the binding mode of this compound highlighted that the 6-ethyl in the α-position could establish hydrophobic contacts with P384 and Y369, positioned at a slightly longer distance than the optimal admitted for VdW interactions. However, it should be noted that the docking approach considers the protein receptor as rigid and didn't allow for mutual adaptation, which is an important process in ligand-receptor binding. In agreement with docking results, the lower efficacy observed for BAR502 could be explained with a slight change in the binding mode, with a different position of the compound in the pocket in order to allow the hydroxyl group on a shortened side chain to interact with the side chain hydroxyl group of T376.

Moreover, also the comparison of the binding modes for G-CDCA and G-UDCA supported the hypothesis that the main determinant for the activity should be related to the network of hydrophobic interactions more than to the lack of a punctual hydrogen bond. Indeed, unlike the weakly active UDCA, the steroid core of G-UDCA is shifted to T376, and the resulting binding mode looks very similar to G-CDCA's. Finally, the better inhibitory efficacy of BAR501 with respect to UDCA, further confirmed the not-essential effect of the charged group on the side chain in terms of inhibition activity. Interestingly, the analysis of the binding mode of BAR501 also suggested that the stereochemistry of the ethyl group at C-6 is not pharmacophoric, being the 6β-ethyl group still able to potentially interact with P384 and Y369.

In the present study, we have developed a strategy to target the interaction of SARS-CoV-2 S protein RBD with the ACE2 receptor. As described in the introduction, SARS-CoV-2 enters the target cells by binding the carboxypeptidase domain of the ACE2 receptor, exposing a cleavage site, a hinge region between S1 and S2, to TMPRSSS2, which in turn allows the S2 subunit of the Spike protein to bind with the cell membrane, leading to the virus/host cells membrane fusion and SARS-CoV-2 penetration in to host cells.

The two pockets we have identified in the β-sheet core of the Spike RBD appear to be targetable by steroidal molecules and, importantly, we found that both naturally occurring bile acids and their metabolites in humans reduce the binding of Spike's RBD to ACE2. Of interest, natural bile acids, such as UDCA, T-UDCA, CDCA, and G-CDCA, exerted a greater inhibitory effect when preincubated with Spike reaching ~45-50% of binding inhibition. Importantly, we found that most of the agents tested in this study were agonists of two main bile acid activated receptors, i.e., the Farnesoid-x-Receptor (FXR) and a cell membrane receptor known as GPBAR1. Thus, betulinic acid and oleanolic acid, along with UDCA and its metabolites, BAR501 and BAR502 are effective ligands for GPBAR1. In contrast, glycyrrhetinic acid, CDCA, G-CDCA and T-CDCA, OCA and BAR704 are known for their ability to bind FXR (Festa et al., 2014). The fact that FXR/GPBAR1agonists bind the SARS-CoV-2 RBD is of general interest and deserve further investigations.

Of interest, some of these agents have been reported for the potential use as anti-HIV agents (Rezanka et al., 2009), and oleanolic acid has been reported as a broad spectrum entry inhibitor of influenza viruses (Yang et al., 2018). On the other side, betulinic acid has been demonstrated to be useful in reducing inflammation and pulmonary edema induced by influenza virus (Hong et al., 2015), and potassium canrenoate, the main metabolite of spironolactone *in vivo*, is an anti-aldosteronic/diuretic used in the treatment of hypertensive patients. Finally, several GPBAR1 and FXR ligands, as bile acid derivatives, have been proved to exert beneficial effects in immune disorders (Fiorucci et al., 2018) and among these, BAR501, the first example of a C-6β-substituted UDCA derivative with potent and selective GPBAR1 activity, has been recently demonstrated as a promising lead in attenuating inflammation and immune dysfunction by shifting the polarization of colonic macrophages from the inflammatory phenotype M1 to the anti-inflammatory phenotype M2, increasing the expression of IL-10 gene transcription in the intestine and enhanced secretion of IL-10 by macrophages (Biagioli et al., 2017).

One important observation we have made in this study is that, while two different pockets of Spike RBD are potentially druggable, these are contiguous, and indeed, when we attempted drug combinations, none of these combinations effectively increased the anti-adhesive efficacy in comparison to the single agent.

This study has several limitations. First of all, we observed that the anti-adhesive efficacy of hyperimmune plasmas obtained from donors who have recovered from COVID-19 and containing high titles of neutralizing antibodies, in inhibiting the Spike RBD/ACE2 interaction, is close to 99%. This percentage is significantly higher than what we measured with our compounds. One possible explanation of this different efficacy can be found in terms of difference in affinity of our compounds with respect to the antibodies but could also be related to the mechanism of allosteric connections suggested by dynamical network and community map analysis. Indeed pockets 1 and 5 resulted tightly connected with the loop G496-Y505, and weakly with the larger loop F456-F490. This suggests that small molecules binding the hydrophobic pockets are less effective than a neutralizing antibody. This also suggests that our pharmacological approach will likely be poorly effective in the presence of a high viral load, and the approach we have developed might have some efficacy only in the case of low viral load. Nevertheless, the mild inhibition efficacy showed by bile acids and their derivatives could pave the way for a further optimization of the binding mode in order to identify additional potential interactions, particularly in pocket 5, which has been demonstrated the least exposed to mutations.

Another limitation is that we have not tested the effect of these treatments on viral replication and further studies are needed to clarify this point.

In conclusion, in this paper, we report the identification of several potential binding sites in the RBD of the SARS-CoV-2 S protein. Several triterpenoids, such as glycyrrhetinic and oleanolic acids, and natural bile acids and their semisynthetic derivatives have been proven effective in reducing the Spike RBD's adhesion to its ACE2 consensus *in vitro*. Altogether, these results might help to define novel approaches to COVID-19 by using SARS-CoV-2 entry inhibitors.

## Data Availability Statement

The raw data supporting the conclusions of this article will be made available by the authors, without undue reservation.

## Ethics Statement

Ethical review and approval was not required for the study on human participants in accordance with the local legislation and institutional requirements. The patients/participants provided their written informed consent to participate in this study.

## Author Contributions

SB and DF provided serum samples. BF, FM, and BC performed virtual screening and analyzed the data. CF and VS performed chemical synthesis. AC, SM, and MB generated the *in vitro* data and performed the data analysis. AZ, BC, ED, and SF conceived the study. All authors drafted the manuscript and wrote the final submission.

## Conflict of Interest

This paper was supported by a research grant by BAR Pharmaceuticals S.r.L. to the Department of Pharmacy of the University of Napoli Federico II and to the Department of Surgical and Biomedical Sciences, University of Perugia. The authors declare the following competing financial interest(s): SF, AZ, and BC have filed an Italian patent application no.102020000011092 in the name of BAR Pharmaceuticals S.r.L. on the compounds described in this paper. The remaining authors declare that the research was conducted in the absence of any commercial or financial relationships that could be construed as a potential conflict of interest.
